# Existing capacity for renal replacement therapy and site-specific practices for managing acute kidney injury at centers participating in the BaSICS trial

**DOI:** 10.5935/0103-507X.20180058

**Published:** 2018

**Authors:** Fernando Godinho Zampieri, Flavio Araújo, Renato Hideo Nakagawa Santos, Alexandre Biasi Cavalcanti

**Affiliations:** 1 Instituto de Pesquisa, HCor-Hospital do Coração - São Paulo (SP), Brasil.

**Keywords:** Acute kidney injury, Renal replacement therapy, Surveys and questionnaires, Intensive care units

## Abstract

**Objective:**

To investigate the existing capacity for renal replacement therapy and site-specific practices for managing acute kidney injury at centers participating in the BaSICS trial.

**Methods:**

A questionnaire was provided to the chairs of 61 intensive care units enrolled in a randomized clinical trial in Brazil. A total of 124 physicians completed the questionnaire.

**Results:**

Approximately 15% of the patients admitted to the analyzed intensive care units received renal replacement therapy at the time of data collection. At least one renal replacement method was available in all of the analyzed units. Continuous methods were available more frequently at the private units than at the public units. The time from indication to onset of treatment was longer at the public units than at private units. The main obstacles to treatment initiation at public intensive care units were related to the availability of equipment and personnel, while the main bottleneck at private units was the nephrologist assessment. A considerable proportion of the participants stated that they would change their approach to renal replacement therapy if there were no limitations on the availability of methods in their units.

**Conclusion:**

There was wide variation in the availability of resources for renal replacement therapy and in the management of acute kidney injury in Brazilian intensive care units. This information should be taken into account when planning clinical trials focused on this topic in Brazil.

## INTRODUCTION

Acute kidney injury (AKI) is a frequent complication among inpatients.^(1)^ According to estimates, up to 16% of inpatients may develop AKI, and the rate of AKI development may be as high as 50% among the critically ill, depending on the definition applied and the population considered.^(1,2)^ Renal replacement therapy (RRT) is potentially lifesaving for severe cases of AKI.

Although having an RRT method available is mandatory for all intensive care units (ICU) in Brazil,^(3)^ AKI management is highly heterogeneous.^(4)^ In addition to personal preferences, the availability of equipment and suitably trained personnel are factors that can interfere with decision-making with regard to AKI. Information on the availability of these resources in Brazilian ICUs is scarce.

The aim of the present study was to assess the operational capacity to start RRT at centers participating in BaSICS (Balanced Solutions in Critical Care Study),^(5)^ including information on the availability of equipment, ICU structure and the routine for ordering RRT at these centers. In addition, data on the management of three hypothetical clinical situations are discussed.

## METHODS

### Participating centers

The 102 Brazilian ICUs that consented to participate in BaSICS^(5)^ were invited to complete a questionnaire (Appendix 1). All unit chairs were asked to respond to the questionnaire. In addition, intensivists (on both regular and shift schedules) and nephrologists were expected to complete the questionnaire when possible. Responses were anonymous, and ICUs were not identified for the purpose of analysis.

### Questionnaire structure

The questionnaire (Appendix 1) had four sections: ICU data (funding, number of beds and occupancy rate); available RRT resources (equipment, methods, number of patients under RRT at the time of data collection, capacity to perform simultaneous RRT, and routine for RRT indication); respondents' opinion on the various known RRT methods (hemodynamic impact and fluid removal capacity); and presentation of three clinical cases:

- Case 1 - a patient with cardiorenal syndrome and a poor response to furosemide (positive furosemide stress test^(6)^)
- Case 2 - a patient with refractory septic shock, Kidney Disease: Improving Global Outcomes (KDIGO) grade 3^(7)^ and dialysis urgency
- Case 3 - a patient with septic shock and kidney dysfunction (KDIGO grade 3) but without dialysis urgency

For cases 1 and 2, respondents were instructed to indicate what treatment measures they would employ and whether they would choose these measures given a scenario characterized by limitless resources at their unit. Our intention here was to establish whether the ability to perform RRT was limited by technical issues in the units. Although we had also planned to evaluate concordance in the management of cases between nephrologists and intensivists, this analysis could not be performed because the number of nephrologists who completed the questionnaire was too small. With regard to case 3, we asked the participants whether or not they would recommend RRT for the patient. When the response was negative, we asked for the respondent's opinion on which criteria most clearly indicate the need to start RRT.

We chose to focus the questionnaire on the characteristics of centers rather than on individual participants because there are existing Brazilian data on the latter subject.^(4)^

### Data analysis

We subjected the questionnaire data to descriptive analysis. In addition, bar graphs and spider plots were generated. All analyses were performed with R software, version 3.4.3 (Kite-Eating Tree).^(8)^ We chose not to describe null hypothesis rejection tests in the presentation of the data but prioritized a descriptive and probabilistic analysis that was consistent with the sample size. For the variables "availability of continuous methods" and "time to onset of treatment," we performed simple Bayesian analysis. This type of analysis is advantageous because it allows incorporation of the beliefs established *a priori* by the investigators (Bayesian priors) to the collected data, resulting in a posterior distribution of probabilities. Priors can be obtained based on a literature review, previous data, or-when previous data are not available (as was the case of the questionnaire administered)-from the impressions of a group of investigators. Priors can be established from the calculation of median values and specific percentiles. The posterior distribution of probability results from the combination of initial beliefs and obtained data.

For the variable "availability of continuous methods," we defined priors based on beta functions, considering a median availability of continuous methods of 25% (90^th^ percentile of 60%), 50% (90^th^ percentile of 75%) and 75% (90^th^ percentile of 90%) for public, mixed and private ICUs, respectively. Additionally, for the variable "time to onset of dialysis," we established priors based on beta functions, considering a median time to initiate RRT of over 4 hours of 60% (90^th^ percentile of 80%), 50% (90^th^ percentile of 75%) and 40% (90^th^ percentile of 50%) for public, mixed and private ICUs, respectively. The 90% probability interval was calculated from 1,000 posterior beta distribution samples.

The corresponding beta functions were calculated using the LearnBayes package. We used the data obtained to update our priors and thus establish the posteriors. The results are presented as a triplot graph, which includes the distribution of sample priors, likelihood and posteriors. The priors were based on the expectation that the availability of continuous methods and time to onset of treatment would be greater in the public ICUs than in private ICUs.

## RESULTS

A total of 124 questionnaires were returned. Sixty-one were completed by ICU chairs (50%), 35% by physicians who worked regular hours (28.2%), 10 by physicians on a shift schedule (8.1%) and 17 by nephrologists (13.7%). Sixty-one valid responses from unit chairs were included in the analysis of ICU characteristics (Table 1). The bed occupancy rate was higher at the public ICUs, which also had waiting lists for admission (more than 4 days per week in approximately 80% of the services).

**Table 1:** Characteristics of the included units

	Funding source			
	Public (n = 24)	Mixed (n = 18)	Private (n = 19)	Total (n = 61)
Active beds	19 [9 - 24]	19 [10 - 20]	30 [20 - 40]	20 [10 - 30]
What is the usual occupancy rate?				
> 90%	18/24 (75)	12/18 (66.7)	6/19 (31.6)	36/61 (59)
70% - 90%	5/24 (20.8)	5/18 (27.8)	12/19 (63.2)	22/61 (36.1)
50% - 70%	1/24 (4.2)	1/18 (5.6)	1/19 (5.3)	3/61 (4.9)
< 50%	0/24 (0)	0/18 (0)	0/19 (0)	0/61 (0)
I don’t know	0/24 (0)	0/18 (0)	0/19 (0)	0/61 (0)
Monthly admissions				
< 20	2/24 (8.3)	0/18 (0)	0/19 (0)	2/61 (3.3)
20 - 40	8/24 (33.3)	3/18 (16.7)	1/19 (5.3)	12/61 (19.7)
40 - 60	6/24 (25)	4/18 (22.2)	2/19 (10.5)	12/61 (19.7)
60 - 80	2/24 (8.3)	2/18 (11.1)	3/19 (15.8)	7/61 (11.5)
80 - 100	2/24 (8.3)	1/18 (5.6)	0/19 (0)	3/61 (4.9)
> 100	4/24 (16.7)	8/18 (44.4)	13/19 (68.4)	25/61 (41)
I don’t know	0/24 (0)	0/18 (0)	0/19 (0)	0/61 (0)
Number of patients currently admitted	17.5 [9 - 22.2]	16.5 [10 - 20]	27 [17.5 - 37]	18 [10 - 27]
Is there a list of patients waiting for an ICU bed?				
Yes, most days (more than 4 days in a regular week)	19/24 (79.2)	12/18 (66.7)	3/19 (15.8)	34/61 (55.7)
Yes, less than half of the week (3 or fewer days per week)	3/24 (12.5)	1/18 (5.6)	7/19 (36.8)	11/61 (18)
Seldom (1 day per week maximum)	1/24 (4.2)	3/18 (16.7)	9/19 (47.4)	13/61 (21.3)
Never	1/24 (4.2)	1/18 (5.6)	0/19 (0)	2/61 (3.3)
I don’t know	0/24 (0)	1/18 (5.6)	0/19 (0)	1/61 (1.6)

ICU - intensive care unit. The results are expressed as the mean [median] or n/n total (%).

**Figure 1:**
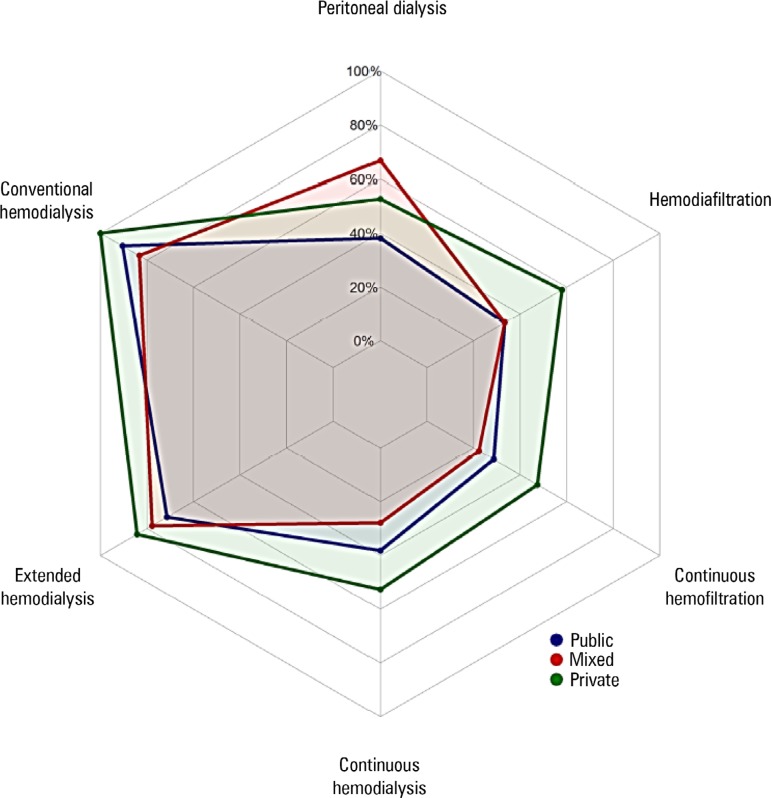
Availability of renal replacement therapy methods according to funding source (n = 61).

**Figure 2:**
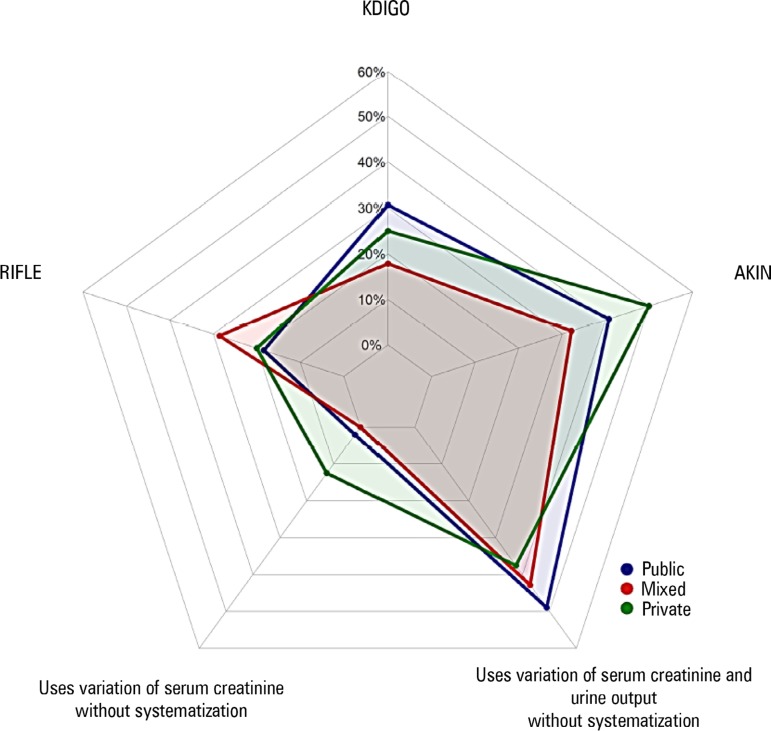
Approaches to acute kidney injury diagnosis. More than one method might be applied at each unit (n = 61). KDIGO - Kidney Disease Improving Global Outcomes; AKIN - Acute Kidney Injury Network; RIFLE - Risk, Injury, Failure, Loss, and End-Stage Renal Failure.

The available resources for RRT are described in table 2. There were differences between the public and private ICUs in several of the measures relating to available resources and capacity. The availability of continuous methods was lower in public ICUs (Table 2 and Figure 1). In the public ICUs, RRT was performed by technicians more often than nurses: RRT was performed by nurses specialized in nephrology in only 19% of such services. At the time of data collection, 21.4% of the patients in public ICUs received some form of RRT compared to 11.1% in private ICUs. The maximum RRT capacity (i.e., the highest percentage of patients receiving RRT simultaneously) was close to 20% in all three types of analyzed ICUs. Nonsystematized creatinine assessment (i.e., creatinine level without specification of the Acute Kidney Injury Network-AKIN, KDIGO, or Risk, Injury, Failure, Loss, and End-Stage Renal Failure-RIFLE criteria) and urine output were the criteria most frequently applied for the diagnosis of AKI. The KDIGO and AKIN scales were used in approximately 30% of cases (Figure 2).

**Table 2:** Existing capacity for renal replacement therapy

	Funding source			
	Public (n = 24)	Mixed (n = 18)	Private (n = 19)	Total (n = 61)
Which renal replacement therapy methods is your unit able to provide?				
Conventional hemodialysis	19/21 (90.5)	15/18 (83.3)	19/19 (100)	53/58 (91.4)
Extended hemodialysis	15/21 (71.4)	14/18 (77.8)	16/19 (84.2)	45/58 (77.6)
Continuous hemodialysis	8/21 (38.1)	5/18 (27.8)	10/19 (52.6)	23/58 (39.7)
Continuous hemofiltration	6/21 (28.6)	4/18 (22.2)	9/19 (47.4)	19/58 (32.8)
Hemodiafiltration	7/21 (33.3)	6/18 (33.3)	11/19 (57.9)	24/58 (41.4)
Peritoneal dialysis	8/21 (38.1)	12/18 (66.7)	10/19 (52.6)	30/58 (51.7)
None	0/21 (0)	0/18 (0)	0/19 (0)	0/58 (0)
How many patients receiving continuous renal replacement therapy can your unit assist simultaneously?	1 [1 - 2.2] (n = 12)	2 [1.5 - 3.5] (n = 7)	4.5 [2.5 - 5.2] (n = 12)	2 [1 - 4] (n = 31)
Proportion (patients/beds)	11.8 [6.4 - 15.7] (n = 12)	10 [9 - 17.4] (n = 7)	12.9 [9.8 - 16.3] (n = 12)	12.5 [8.2 - 17.2] (n = 31)
How many patients receiving intermittent renal replacement therapy can your unit assist simultaneously?	3.5 [2 - 5] (n = 20)	2.5 [2 - 5] (n = 16)	5 [3 - 6.5] (n = 19)	4 [2 - 6] (n = 55)
Proportion (%) (patients/beds)	23.2 [13.1 - 28.7] (n = 20)	15.8 [10.8 - 33.1] (n = 16)	20 [10 - 21.6] (n = 19)	20 [11.1 - 28.2] (n = 55)
How many patients receiving renal replacement therapy of any kind can your unit assist simultaneously?	3 [2 - 4] (n = 21)	2.5 [2 - 4.8] (n = 18)	6 [3 - 8] (n = 19)	3 [2 - 5.8] (n = 58)
Proportion (patients/beds)	20 [12.5 - 33.3] (n = 21)	20 [11.1 - 34.5] (n = 18)	20 [11.8 - 23.1] (n = 19)	20 [11.1 - 29] (n = 58)
How many patients receive renal replacement therapy in a typical month?				
< 5% of patients	0/21 (0)	3/18 (16.7)	2/19 (10.5)	5/58 (8.6)
5% - 10% of patients	7/21 (33.3)	3/18 (16.7)	7/19 (36.8)	17/58 (29.3)
10% - 20% of patients	7/21 (33.3)	8/18 (44.4)	7/19 (36.8)	22/58 (37.9)
> 20% of patients	7/21 (33.3)	3/18 (16.7)	1/19 (5.3)	11/58 (19)
I don’t know	0/21 (0)	1/18 (5.6)	2/19 (10.5)	3/58 (5.2)
Right now, how many patients are receiving some form of renal replacement therapy?	3 [2 - 4] (n = 21)	3 [1 - 3.8] (n = 18)	4 [2 - 5] (n = 19)	3 [2 - 4] (n = 58)
Proportion (patients/beds)	21.4 [12.5 - 25] (n = 21)	15 [9.1 - 17.7] (n = 18)	11.1 [7 - 15] (n = 19)	15 [8.8 - 21.4] (n = 58)
Which professionals operate the renal replacement therapy equipment in the ICU?				
ICU nurse with a specialization in nephrology	4/21 (19)	3/18 (16.7)	6/19 (31.6)	13/58 (22.4)
A nurse from the hospital dialysis center assigned to the ICU	6/21 (28.6)	9/18 (50)	12/19 (63.2)	27/58 (46.6)
ICU nursing technician with specific training	9/21 (42.9)	8/18 (44.4)	4/19 (21.1)	21/58 (36.2)
Other	8/21 (38.1)	6/18 (33.3)	4/19 (21.1)	18/58 (31)
How many proportioning devices for conventional or extended renal replacement therapy are available in your unit?				
None	2/21 (9.5)	3/18 (16.7)	1/19 (5.3)	6/58 (10.3)
1	6/21 (28.6)	7/18 (38.9)	3/19 (15.8)	16/58 (27.6)
2	6/21 (28.6)	4/18 (22.2)	5/19 (26.3)	15/58 (25.9)
3	2/21 (9.5)	0/18 (0)	1/19 (5.3)	3/58 (5.2)
4	3/21 (14.3)	1/18 (5.6)	0/19 (0)	4/58 (6.9)
5 or more	2/21 (9.5)	3/18 (16.7)	9/19 (47.4)	14/58 (24.1)
How many machines for extended renal replacement therapy are available in your unit?				
None	15/21 (71.4)	13/18 (72.2)	11/19 (57.9)	39/58 (67.2)
1	1/21 (4.8)	2/18 (11.1)	4/19 (21.1)	7/58 (12.1)
2	4/21 (19)	1/18 (5.6)	2/19 (10.5)	7/58 (12.1)
3	1/21 (4.8)	0/18 (0)	1/19 (5.3)	2/58 (3.4)
4	0/21 (0)	1/18 (5.6)	1/19 (5.3)	2/58 (3.4)
5 or more	0/21 (0)	1/18 (5.6)	0/19 (0)	1/58 (1.7)
How many machines for slow renal replacement therapy are available in your unit?				
None	13/21 (61.9)	11/18 (61.1)	10/19 (52.6)	34/58 (58.6)
1	6/21 (28.6)	3/18 (16.7)	3/19 (15.8)	12/58 (20.7)
2	2/21 (9.5)	3/18 (16.7)	0/19 (0)	5/58 (8.6)
3	0/21 (0)	0/18 (0)	1/19 (5.3)	1/58 (1.7)
4	0/21 (0)	0/18 (0)	1/19 (5.3)	1/58 (1.7)
5 or more	0/21 (0)	1/18 (5.6)	4/19 (21.1)	5/58 (8.6)

ICU - intensive care unit. The results are expressed as the mean [median] or n/n total (%).

The process of patient evaluation and RRT initiation differed as a function of the funding source (Table 3). In the private ICUs, the process of evaluation and the decision to start RRT frequently included additional steps; intensivists discussed the need for evaluation by a nephrologist with the attending physicians, and the decision to start RRT was made only following staff consensus (15.8%). This practice was much less common at public ICUs (4.8%).

**Table 3:** Process of renal replacement therapy indication and treatment initiation

	Funding source			
	Public (n = 24)	Mixed (n = 18)	Private (n = 19)	Total (n = 61)
Which process best represents the approach for starting renal replacement therapy in your unit?				
An intensivist establishes the diagnosis of acute kidney injury and indicates the need for replacement therapy. A nephrologist prescribes renal replacement therapy.	2/21 (9.5)	3/18 (16.7)	1/19 (5.3)	6/58 (10.3)
An intensivist establishes the diagnosis of acute kidney injury and requests assessment by a nephrologist. The nephrologist determines the indication and prescribes renal replacement therapy.	18/21 (85.7)	10/18 (55.6)	15/19 (78.9)	43/58 (74.1)
An intensivist establishes the diagnosis of acute kidney injury and discusses with the attending physician the need for assessment by a nephrologist. The nephrologist is called and discusses with the staff the need for renal replacement therapy. The nephrologist prescribes renal replacement therapy.	1/21 (4.8)	2/18 (11.1)	3/19 (15.8)	6/58 (10.3)
The ICU staff includes a nephrologist who is in charge of the assessment and follow-up of patients with acute kidney injury and prescribes replacement therapy as needed.	0/21 (0)	3/18 (16.7)	0/19 (0)	3/58 (5.2)
What is the average time from indication to initiation of renal replacement therapy?				
< 2 hours	3/21 (14.3)	7/18 (38.9)	4/19 (21.1)	14/58 (24.1)
2 - 4 hours	4/21 (19)	4/18 (22.2)	12/19 (63.2)	20/58 (34.5)
4 - 6 hours	6/21 (28.6)	1/18 (5.6)	3/19 (15.8)	10/58 (17.2)
> 6 hours	8/21 (38.1)	5/18 (27.8)	0/19 (0)	13/58 (22.4)
I don’t know	0/21 (0)	1/18 (5.6)	0/19 (0)	1/58 (1.7)
What is the limiting step in the process of starting renal replacement therapy once it is prescribed?				
Nephrologist assessment	0/21 (0)	1/18 (5.6)	7/19 (36.8)	8/58 (13.8)
Bureaucracy (e.g., payer’s authorization)	0/21 (0)	0/18 (0)	0/19 (0)	0/58 (0)
Equipment availability	7/21 (33.3)	11/18 (61.1)	1/19 (5.3)	19/58 (32.8)
Availability of personnel to start the procedure	10/21 (47.6)	4/18 (22.2)	5/19 (26.3)	19/58 (32.8)
Adequate vascular access	4/21 (19)	2/18 (11.1)	6/19 (31.6)	12/58 (20.7)
Other	0/21 (0)	0/18 (0)	0/19 (0)	0/58 (0)

ICU - intensive care unit. The results are expressed as n/n total (%).

The time from indication to onset of RRT was greater than 6 hours in more than one-fifth of the analyzed ICUs. The reasons for the delay varied as a function of the funding source. In public ICUs, the delay was often due to the unavailability of equipment or personnel to start the procedure (33.3% and 47.6%, respectively). The evaluation by the nephrologist was the main cause of delay in the private ICUs (38.6%).

**Figure 3:**
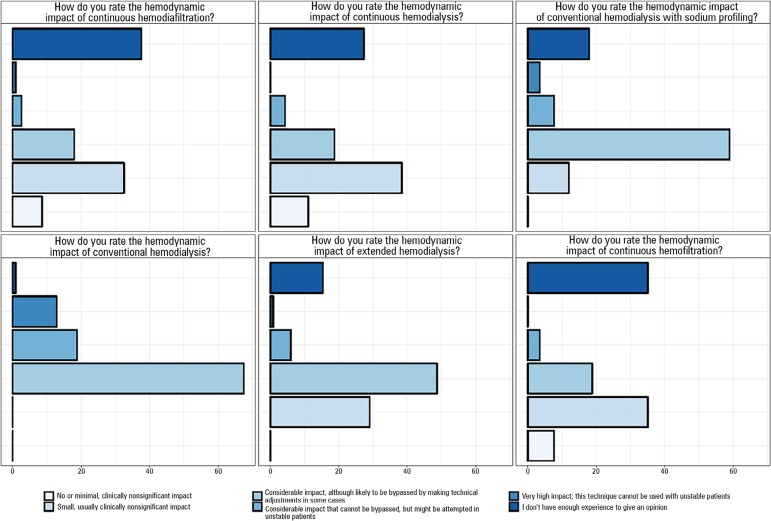
Respondents' opinion (n = 124) of the hemodynamic impact of each renal replacement therapy method.

With regard to participants' subjective impression of the impact of RRT methods, most respondents observed that conventional methods of dialysis are associated with a significant hemodynamic impact, which could potentially be circumvented by making technical adjustments. Overall, the continuous methods were described as having less of an impact (absent or small, and clinically insignificant); however, some respondents stated that the impact is clinically significant. A considerable number of participants did not provide an opinion on the continuous methods due to lack of experience (Figure 3).

**Figure 4:**
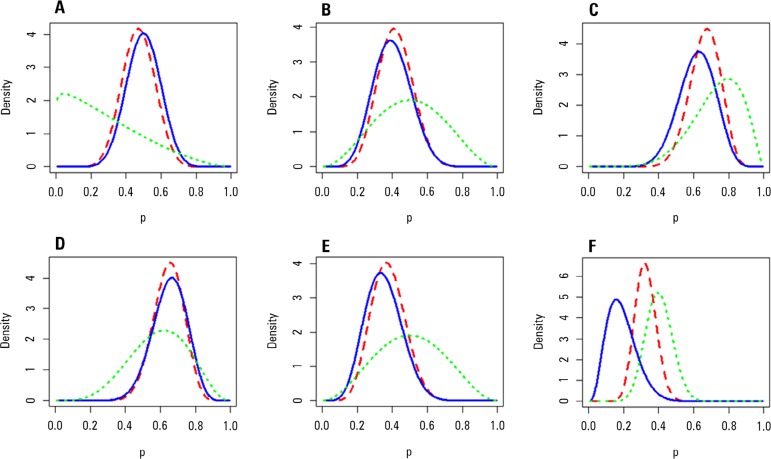
Distribution of priors (green), likelihood (blue) and posteriors relative to availability of continuous methods at intensive care units (graphs A, B and C respectively correspond to public, mixed and private intensive care units) and more than 4 hours from indication to onset of treatment (graphs D, E and F respectively correspond to public, mixed and private intensive care units).

The results of Bayesian analysis of the availability of continuous methods and time to onset of RRT are described in figure 4. For the availability of continuous methods, the 90% probability interval was 32%-62% for public ICUs, 26%-59% for ICUs with mixed funding, and 52%-80% for private ICUs. For a delay of over 4 hours until the start of RRT, the probability interval was 49%-79% for public ICUs, 23%-55% for ICUs with mixed funding, and 22%-43% for private ICUs.

The measures selected for each assessed clinical case are described in table 4. For case 1, 49.9% of the sample indicated the need for conservative management, and 50.4% recommended some form of RRT (a continuous method according to 11.3% of the respondents). Thirty-two of 115 respondents stated that they would change their choice, with a trend to prefer extended or continuous RRT. For case 2, all of the participants indicated the need to start RRT, and most indicated the need to use extended or continuous methods. When 46 respondents were asked whether they would change their choice in the absence of restrictions on equipment or personnel on the service, most stated that they would choose a continuous method. For case 3, most participants (80.9%) responded they would start RRT. Oliguria and positive fluid balance were described as the most pressing indicators for RRT.

**Table 4:** Measures taken for the described cases

**Clinical case 1**	
Considering the measures usually adopted in your unit and the site limitations, what would you do?	
An additional dose of furosemide; consider continuous furosemide IV	57/115 (49.6)
Start intermittent hemodialysis	17/115 (14.8)
Start extended hemodialysis	28/115 (24.3)
Start continuous renal replacement therapy	13/115 (11.3)
Would change measures in case of no limitations	32/115 (27.8)
What would you choose ?	
Start intermittent hemodialysis	3/31 (9.7)
Start extended hemodialysis	9/31 (29)
Start continuous renal replacement therapy	17/31 (54.8)
Other (hemofiltration)	2/31 (6.5)
**Clinical case 2**	
Considering the measures usually adopted in your unit and the site limitations, what would you do?	
Diuretic drug	0/115 (0)
Hydration	0/115 (0)
Start intermittent hemodialysis	31/115 (27)
Start extended hemodialysis	41/115 (35.7)
Start continuous renal replacement therapy	43/115 (37.4)
Would change measures in case of no limitations	46/115 (40)
What would you choose?	
Start intermittent hemodialysis	0/46 (0)
Start extended hemodialysis	5/46 (10.9)
Start continuous renal replacement therapy	40/46 (87)
Other	1/46 (2.2)
**Clinical case 3**	
Would you indicate renal replacement therapy for this patient?	93/115 (80.9)
For this patient, what would be the primary indicator to start renal replacement therapy provided that all other variables remain constant?	
Serum potassium	17/115 (14.8)
What is the cutoff point to indicate dialysis?	6 [6 - 7] (n = 17)
Oliguria	45/115 (39.1)
What is the minimum 12-hour urine output that contraindicates renal replacement therapy in a 70kg patient?	420 [400 - 450] (n = 45)
pH	9/115 (7.8)
Serum pH below which value?	7.2 [7.2 - 7.2] (n = 9)
Positive fluid balance	32/115 (27.8)
Starting at how many liters of cumulative fluid?	5 [3 - 7.2] (n = 32)
Serum urea	5/115 (4.3)
G7, Starting at which serum urea level, in mg/dL	150 [80 - 200] (n = 5)
Uremia symptoms	7/115 (6.1)
Which uremia symptom?	
Blood disorders	1/7 (14.3)
Uremic encephalopathy	6/7 (85.7)
Nausea	0/7 (0)

The results are expressed as n/n total (%) and mean [median].

## DISCUSSION

The questionnaire responses collected from 124 physicians at 61 Brazilian ICUs provided relevant data on the availability of resources for RRT in Brazil. Our data point to divergences between public and private services in several aspects, including the number of beds, the occupancy rate and the existence of waiting lists for admission to the ICU. Information on waiting lists is seldom reported and indicates a patient overload and a shortage of beds in public units.

Although all of the analyzed units have the ability to perform RRT, we detected considerable differences in the approach used to diagnose and manage AKI. The application of the KDIGO and AKIN scales notwithstanding, nonsystematized creatinine and urine output assessments are still frequently performed at the analyzed ICUs. The unit chairs reported that approximately 15% of the patients were receiving RRT at the time of data collection. This rate was higher than that at the public units (21.4% *versus* 11.1%). Considering that the maximum reported capacity was 20%, on average, we might infer that the public ICUs are operating at close to their maximum capacity to provide RRT, while the private units had some technical reserve. However, this conclusion should be interpreted cautiously, and the differences in patient severity that are usually observed between public and private ICUs should be taken into account. Considering the frequent existence of waiting lists for admission to public ICUs, our data corroborate the idea that this type of service operates under overt strain.^(9)^ This situation might partially account for the poorer outcomes usually reported for public ICUs in Brazil.^(10)^ In addition, operational factors might influence the decision to admit a critically ill patient to the ICU,^(11)^ which may further complicate the analysis of outcomes.

The process of initiating RRT also differed based on the ICU profiles. The decision to start RRT was frequently more direct at the public ICUs, possibly because these ICUs operate under a closed system in which intensivists are directly responsible for most decisions. In contrast, the open system was the most common at private ICUs, the decision to start RRT was often more thoroughly discussed, and the process included some additional steps (such as consulting the attending physician on the need to call a nephrologist).

Thus, it was not surprising that the bottlenecks in the path to RRT initiation were quite different between the two types of ICUs. The limiting factors at public ICUs operating close to their maximum capacity were the availability of equipment and personnel. On the other hand, the time until the nephrologist evaluated the patient was a limiting factor at private ICUs. In 38.1% of the public ICUs, the time from indication to onset of RRT was over 6 hours. The ICUs with mixed funding exhibited intervals that were intermediate between those of the public and private units in most analyses. Graphs C, D and E in Figure 4 represent the *a posteriori* distribution of the probability (together with the priors used and the calculated likelihood) of RRT being effectively initiated more than 4 hours after indication. Indeed, the graphs show a gradient between the public and the private ICUs.

Most respondents indicated that intermittent methods have a considerable hemodynamic impact, which could potentially be bypassed by making technical adjustments. Indeed, strategies such as using cold dialysate and sodium profiling might improve the hemodynamic tolerance to the procedure.^(12,13)^ However, an analysis of the responses to the clinical cases showed that for a considerable proportion of the time, only the conventional method was available, but the respondents would have chosen a continuous method if available. In case 1 in particular, the availability of continuous methods, or increased accessibility of intermittent methods, would have made the participants change their decision on the treatment method. Therefore, limitations in resources might influence the clinical decision with regard to the binary outcome usually considered in clinical trials (onset of RRT).^(14)^ This finding highlights the importance of assessing the operational capacity of participating centers before defining outcomes for clinical trials. Upon analyzing the *a posteriori* distribution of the availability of continuous methods according to the funding source (graphs A, B and C in Figure 4), we were favorably surprised by the availability of continuous methods at public ICUs-much greater than the established prior. However, the large 90% probability intervals in this analysis and the small number of responses indicate the need for caution in the interpretation of these data.

The present study has several limitations. The first limitation derives from the method for the selection of the participating units. BaSICS sought to include the largest possible number of Brazilian ICUs, and for this reason, no exclusion criteria were established. The invitation to participate was distributed through various media, including the e-mail lists of several institutions [such as *Instituto Latino Americano da Sepse* (Latin American Institute of Sepsis - ILAS) and the *Rede Brasileira de Pesquisa em Terapia Intensiva* (Brazilian Network for Intensive Care Research - BRICNet)], social networks and instant messaging groups. In addition, the questionnaire was distributed to the participants of the first meeting of investigators held during the XXI Brazilian Congress of Intensive Medicine, Porto Alegre (2016). Despite wide dissemination of the questionnaire, the final sample consisted of only those units that agreed to participate in the study and thus may not accurately represent the profile of ICUs across Brazil. Therefore, the inferences drawn from Bayesian analysis are applicable to the population of ICUs eligible for the study but not to all Brazilian ICUs. We were unable to compare the measures selected for the clinical cases as a function of the respondents' field of activity due to the small number of responses from some groups. We sought to describe the cases in the survey in as much detail as possible; however, we might have omitted details relevant to some participants. The anonymous nature of the responses did not allow us to evaluate regional characteristics of the participating centers. Even in instances when the unit chairs designated a colleague from the same service to respond the questionnaire, the relationship of these individuals was not reported to the study coordination center. As a result, we could not assess the degree of concordance in the measures among respondents from the same center. Finally, we emphasize that the sample was small, and thus, the results should be interpreted cautiously. As with any questionnaire, the information collected reflects the impressions of the respondents and thus is subject to imprecision.

## CONCLUSION

There was wide variation in the availability of resources for renal replacement therapy and in the management of acute kidney injury among Brazilian intensive care units, with considerable differences between public and private units. This information should be taken into account when planning clinical trials targeting this subject in Brazil.

## Appendix 1

**Table:** Questionnaire

Use of renal replacement therapy in the ICU. A survey of BaSICS study participants
This questionnaire comprises 53 questions

[ ] What is your function in the unit?
Please select only one of the following options:
○ ICU chair
○ ICU physician (regular work schedule)
○ ICU physician (shift schedule)
○ Hospital nephrologist

[ ] Are you also a physician who works regular hours or shifts at the unit where you perform dialysis?
**Answer this question only if:**
The response to question ‘1 [type]’ (What is your function in the unit?) was ‘hospital nephrologist’
Please select only one of the following options:
○ Yes
○ No

**ICU data**
[ ] What is the funding source for your unit?
Please select only one of the following options:
○ Public
○ Mixed, predominantly public
○ Mixed, predominantly private
○ Private

[ ] How many active ICU beds are there in your unit?
Please fill the box using numbers only.
Please write your answer here:

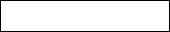

[ ] What is the usual bed occupancy rate?
Please select only one of the following options:
○ Over 90%
○ 70 to 90%
○ 50 to 70%
○ Less than 50%
○ I don’t know

[ ] How many patients are admitted to your unit per month, on average?
Please select only one of the following options:
○ Fewer than 20
○ 20 to 40
○ 40 to 60
○ 60 to 80
○ 80 to 100
○ More than 100
○ I don’t know

[ ] Right now, how many patients are admitted to the ICU?
Please fill the box using numbers only.
Please write your answer here:

[ ] Is there a list of patients waiting for an ICU bed? In other words, is there a waiting list for admission to the ICU?
Please select only one of the following options:
○ Yes, most days (more than 4 days in a regular week)
○ Yes, less than half of the week (3 or fewer days per week)
○ Seldom (1 day per week maximum)
○ Never
○ I don’t know

**Available resources for renal replacement therapy**
[ ] Which renal replacement therapy methods is your unit able to provide (mark all methods applicable)?
Please select all applicable options:
□ Conventional hemodialysis with proportioning system
□ Extended hemodialysis
□ Continuous hemodialysis
□ Continuous hemofiltration
□ Hemodiafiltration
□ Peritoneal dialysis
□ None

[ ] How many patients under continuous renal replacement therapy (“slow” therapy, i.e., hemofiltration, hemodialysis, hemodiafiltration) can your unit assist simultaneously? Mark zero if these therapies are not available in your unit.
Please fill the box using numbers only.
Please write your answer here:

[ ] How many patients on intermittent renal replacement therapy (conventional, including extended therapy) can your unit assist simultaneously? Mark zero if these therapies are not available in your unit.
Please fill the box using numbers only.
Please write your answer here:

[ ] How many patients on renal replacement therapy of any kind (continuous or intermittent therapy) can your unit assist simultaneously? Mark zero if these therapies are not available in your unit.
Please fill the box using numbers only.
Please write your answer here:

[ ] How many patients receive renal replacement therapy in a typical month?
Please select only one of the following options:
○ Less than 5% of patients
○ 5 - 10% of patients
○ 10 - 20% of patients
○ Greater than 20% of patients
○ I don’t know

[ ] Right now, how many patients are receiving some form of renal replacement therapy (continuous or intermittent)? Consider the patients who depend on the device, even if it is currently turned off. For instance, patients who receive classic hemodialysis on Mondays, Wednesdays and Fridays should be included, even if today is not one of the days they receive therapy.
Please fill the box using numbers only.
Please write your answer here:

[ ] Which professional(s) operate(s) the renal replacement therapy equipment in the ICU (mark all applicable options)?
Please select all that apply:
□ ICU nurse with a specialization in nephrology
□ A nurse from the hospital dialysis center assigned to the ICU
□ ICU nursing technician with specific training
□ Other:
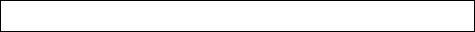

[ ] What is the average time from indication to onset of renal replacement therapy?
Please select only one of the following options:
○ < 2 hours
○ 2 - 4 hours
○ 4 - 6 hours
○ > 6 hours
○ I don’t know

[ ] What is the limiting step (i.e., the one that takes the longest) in the process of initiating renal replacement therapy once it has been prescribed?
If you select “Other,” please elaborate on your response in the box.
Please select only one of the following options:
○ Adequate vascular access
○ Nephrologist assessment
○ Equipment availability
○ Availability of personnel to start the procedure
○ Bureaucracy (e.g., payer’s authorization)
○ Other:

[ ] How many **proportioning devices for classic or extended** (e.g., **Fresenius 4008**) renal replacement therapy are available in your unit?
Please, select only one of the following options:
○ None
○ One (01)
○ Two (02)
○ Three (03)
○ Four (04)
○ Five or more

[ ] How many **SLED (GENIUS)** renal replacement machines are available in your service?
Please select only one of the following options:
○ None
○ One (01)
○ Two (02)
○ Three (03)
○ Four (04)
○ Five or more

[ ] How many **slow (PRISMA FLEX, PRISMA, GAMBRO, etc.)** renal replacement machines are available in your service?
Please, select only one of the following options:
○ None
○ One (01)
○ Two (02)
○ Three (03)
○ Four (04)
○ Five or more

**Usual dialysis practices in the ICU**
[ ] Is a particular diagnostic criterion for acute kidney injury routinely applied in your ICU? Examples: AKIN, RIFLE, KDIGO, etc.
Please select all that apply:
□ KDIGO
□ RIFLE
□ AKIN
□ Serum creatinine variations without systematization
□ Serum creatinine variations and urine output without systematization
□ Other:

[ ] Which process best represents the approach for starting renal replacement therapy in your unit?
Please select only one of the following options:
○ An intensivist establishes the diagnosis of acute kidney injury and indicates the need for replacement therapy. A nephrologist prescribes renal replacement therapy.
○ An intensivist establishes the diagnosis of acute kidney injury and requests assessment by a nephrologist. The nephrologist determines the indication and prescribes renal replacement therapy.
○ An intensivist establishes the diagnosis of acute kidney injury and discusses with the attending physician the need for assessment by a nephrologist. The nephrologist is called and discusses with the staff the need for renal replacement therapy. The nephrologist prescribes renal replacement therapy.
○ The ICU staff includes a nephrologist who is in charge of the assessment and follow-up of patients with acute kidney injury and prescribes replacement therapy as needed.

[ ] Does the **patient’s comfort** influence your decision on which renal replacement method to use in the ICU?
Please select only one of the following options:
○ Not relevant
○ A little relevant
○ Indifferent
○ Relevant
○ Highly relevant

[ ] Does the **risk of osmotic imbalance** influence your decision on which renal replacement method to use in the ICU?
Please select only one of the following options:
○ Not relevant
○ A little relevant
○ Indifferent
○ Relevant
○ Highly relevant

[ ] Do **close electrolyte control and metabolic adjustment** influence your decision on which renal replacement method to use in the ICU?
Please select only one of the following options:
○ Not relevant
○ A little relevant
○ Indifferent
○ Relevant
○ Highly relevant

[ ] Does allowing for the use of **regional anticoagulation** influence your decision on which renal replacement method to use in the ICU?
Please select only one of the following options:
○ Not relevant
○ A little relevant
○ Indifferent
○ Relevant
○ Highly relevant

[ ] Does **hemodynamic tolerance** influence your decision on which renal replacement method to use in the ICU?
Please select only one of the following options:
○ Not relevant
○ A little relevant
○ Indifferent
○ Relevant
○ Highly relevant

[ ] Do **costs** influence your decision on which renal replacement method to use in the ICU?
Please select only one of the following options:
○ Not relevant
○ A little relevant
○ Indifferent
○ Relevant
○ Highly relevant

[ ] Does the **speed of fluid removal** influence your decision on which renal replacement method to use in the ICU?
Please select only one of the following options:
○ Not relevant
○ A little relevant
○ Indifferent
○ Relevant
○ Highly relevant

[ ] Does **availability** influence your decision on which renal replacement method to use in the ICU?
Please select only one of the following options:
○ Not relevant
○ A little relevant
○ Indifferent
○ Relevant
○ Highly relevant

[ ] How do you rate the hemodynamic impact (i.e., the odds for the method to acutely worsen the patient’s hemodynamics) of **conventional hemodialysis**?
Please select only one of the following options:
○ No or minimal, clinically nonsignificant impact
○ Small, usually clinically nonsignificant impact
○ Considerable impact, although likely to be bypassed by making technical adjustments in some cases
○ Considerable impact that cannot be bypassed, but might be attempted in unstable patients
○ Very high impact; this technique cannot be used with unstable patients
○ I don’t have enough experience to provide an opinion

[ ] How do you rate the hemodynamic impact (i.e., the odds for the method to acutely worsen the patient’s hemodynamics) of **conventional hemodialysis with sodium profiling**?
Please, select only one of the following options:
○ No or minimal, clinically nonsignificant impact
○ Small, usually clinically nonsignificant impact
○ Considerable impact, although likely to be bypassed by making technical adjustments in some cases
○ Considerable impact that cannot be bypassed, but might be attempted in unstable patients
○ Very high impact; this technique cannot be used with unstable patients
○ I don’t have enough experience to provide an opinion

[ ] How do you rate the hemodynamic impact (i.e., the odds for the method to acutely worsen the patient’s hemodynamics) of **extended hemodialysis**?
Please, select only one of the following options:
○ No or minimal, clinically nonsignificant impact
○ Small, usually clinically nonsignificant impact
○ Considerable impact, although likely to be bypassed by making technical adjustments in some cases
○ Considerable impact that cannot be bypassed, but might be attempted in unstable patients
○ Very high impact; this technique cannot be used with unstable patients
○ I don’t have enough experience to provide an opinion

[ ] How do you rate the hemodynamic impact (i.e., the odds for the method to acutely worsen the patient’s hemodynamics) of **continuous hemodialysis**?
Please select only one of the following options:
○ No or minimal, clinically nonsignificant impact
○ Small, usually clinically nonsignificant impact
○ Considerable impact, although likely to be bypassed by making technical adjustments in some cases
○ Considerable impact that cannot be bypassed, but might be attempted in unstable patients
○ Very high impact; this technique cannot be used with unstable patients
○ I don’t have enough experience to provide an opinion

[ ] How do you rate the hemodynamic impact (i.e., the odds for the method to acutely worsen the patient’s hemodynamics) of **continuous hemofiltration**?
Please select only one of the following options:
○ No or minimal, clinically nonsignificant impact
○ Small, usually clinically nonsignificant impact
○ Considerable impact, although likely to be bypassed by making technical adjustments in some cases
○ Considerable impact that cannot be bypassed, but might be attempted in unstable patients
○ Very high impact; this technique cannot be used with unstable patients
○ I don’t have enough experience to provide an opinion

[ ] How do you rate the hemodynamic impact (i.e., the odds for the method to acutely worsen the patient’s hemodynamics) of **continuous hemodiafiltration**?
Please select only one of the following options:
○ No or minimal, clinically nonsignificant impact
○ Small, usually clinically nonsignificant impact
○ Considerable impact, although likely to be bypassed by making technical adjustments in some cases
○ Considerable impact that cannot be bypassed, but might be attempted in unstable patients
○ Very high impact; this technique cannot be used with unstable patients
○ I don’t have enough experience to provide an opinion

**Clinical case 1**

A fifty-six-year-old (80kg) patient with a history of congestive heart failure was admitted to the ICU because his dyspnea had worsened the previous week. He exhibits obvious anasarca and has gained approximately 9kg compared to his usual weight. His blood pressure is 90/60mmHg, and his heart rate is 110bpm (with atrial fibrillation). He is well adjusted to bilevel positive airway pressure (BiPAP), expiration pressure 8cmH2O, inspiration pressure 12cmH2O, and requires a fraction of inspired oxygen of 60% to maintain saturation at 90 - 92%. He is comfortable under noninvasive ventilation NIV but needs to use the accessory muscles when breathing spontaneously. He was given a bolus (1mg/kg) of furosemide in the emergency department. Twelve hours following the furosemide dose, the total urine output was only 250mL. Acidosis and hyperkalemia were ruled out. The BNP level is > 5,000, and the serum creatinine us 1.4mg/dL (similar to baseline one month earlier).

[ ] Considering the measures usually adopted in your unit and the site limitations, what would you do?
Please select only one of the following options:
○ Administer an additional dose of furosemide; consider continuous furosemide IV
○ Start intermittent hemodialysis
○ Start extended hemodialysis
○ Start continuous renal replacement therapy

[ ] If there were no technical or personnel limitations in your unit, would you uphold your first choice stated above?
Please select only one of the following options:
○ Yes
○ No

[ ] What would your first choice be in this case?
**Answer this question only if**
You answered ‘No” to question ’38 [C1ConductFull]' (If there was no technical or personnel limitation in your unit, would you uphold your first choice stated above?)
Please select only one of the following options:
○ Start intermittent hemodialysis
○ Start extended hemodialysis
○ Start continuous renal replacement therapy
○ Other:

**Clinical case 2**

A sixty-year-old patient was admitted due to septic shock of abdominal origin (perforated diverticulitis); it is currently his second day in the ICU. He is hemodynamically unstable, even while receiving more than 1mcg/kg/min of norepinephrine. He does not seem to be preload responsive (low pulse pressure variation, dilated vena cava). He exhibits considerable acidosis (base excess 18mEq/L; pH 7.1.) without respiratory participation. The serum potassium is 7.0mEq/L, and all previous efforts to control hyperkalemia have failed. He is currently receiving sodium bicarbonate, 100mEq, every 6 hours. His sodium level is 154mEq/L.

[ ] Considering the measures typically adopted by your service and the site limitations, how would you treat this patient?
Please select only one of the following options:
○ Diuretic drug
○ Hydration
○ Start intermittent hemodialysis
○ Start extended hemodialysis
○ Start continuous renal replacement therapy

[ ] If there were no technical or personnel limitations in your unit, would you uphold your first choice stated above?
Please select only one of the following options:
○ Yes
○ No

[ ] What would your first choice be in this case?
**Answer this question only if**
You answered “No” to question ’41 [C2ConductFull]' (If there were no technical or personnel limitations in your unit, would you uphold your first choice stated above?)
Please select only one of the following options:
○ Start intermittent hemodialysis
○ Start extended hemodialysis
○ Start continuous renal replacement therapy
○ Other

**Clinical case 3**

A sixty-five year old (70kg) patient was admitted to the ICU due to septic shock secondary to severe community-acquired pneumonia (day 3). He is awake, alert and oriented. Oxygenation is good with nasal catheter 3L/min, with borderline saturation (91 - 92%). His blood pressure is 100/60mmHg, and his heart rate is 90 bpm while receiving 0.06mcg/kg/min of norepinephrine (during the past 12 hours). The serum creatinine routinely collected in the morning was 2.5mg/dL (baseline 1.2mg/dL), and the urea level was 120mg/dL. The results of the arterial blood gas test were as follows: normal pH, slightly negative base excess (-4mEq/L; sodium bicarbonate 20mEq/L). The serum potassium is 4.5mEq/L. The urine output was 650mL over the past 12 hours (approximately 0.77mL/kg/h). The cumulative fluid balance is +5L (approximately 7% of the patient’s body weight).

[ ] Would you indicate renal replacement therapy in this patient?
Please, select only one of the following options:
○ Yes
○ No

[ ] For this patient, which of the variables below would you consider the primary indicator for renal replacement therapy provided that all other variables remain constant?
Please select only one of the following options:
○ Serum potassium
○ Oliguria
○ pH
○ Positive fluid balance
○ Serum urea
○ Uremia symptoms

[ ] What is the cutoff point to indicate dialysis?
**Answer this question only if**
You answered “Serum potassium” to question ’44 [C3Reason]' (For this patient, which of the variables below would you consider the primary indicator for renal replacement therapy provided that all other variables remain constant?)
Please fill the box using numbers only.
Please write your answer here:

[ ] What is the minimum 12-hour urinary output that contraindicates renal replacement therapy in a 70-kg patient?
**Answer this question only if**
You answered ‘Oliguria’ to question ’44 [C3Reason]' (For this patient, which of the variables below would you consider the primary indicator for renal replacement therapy provided all other variables remain constant?)
Please fill the box using numbers only.
Please write your answer here:

[ ] Serum pH below which value?
**Answer this question only if**
You answered ‘pH’ to question ’44 [C3Reason]' (For this patient, which of the variables below would you consider the primary indicator for renal replacement therapy provided all other variables remain constant?)
Please fill the box using numbers only.
Please write your answer here:

[ ] Starting at how many liters of cumulative fluid balance?
**Answer this question only if**
You answered ‘Positive fluid balance’ to question ’44 [C3Reason]' (For this patient, which of the variables below would you consider the primary indicator for renal replacement therapy provided that all other variables remain constant?)
Please fill the box using numbers only.
Please write your answer here:

[ ] Starting at which serum urea level, in mg/dL?
**Answer this question only if**
You answered “Serum urea” to question ’44 [C3Reason]' (For this patient, which of the variables below would you consider the primary indicator for renal replacement therapy provided that all other variables remain constant?)
Please fill the box using numbers only.
Please write your answer here:

[ ] Which uremia symptom?
**Answer this question only if**
You answered “Uremia symptoms” to question ’44 [C3Reason]' (For this patient, which of the variables below would you consider the primary indicator for renal replacement therapy provided that all other variables remain constant?)
Please select only one of the following options:
○ Mucosal bleeding
○ Mental confusion
○ Nausea
○ Abdominal pain
○ Other

[ ] If you are an **intensivist**, which variable do you think a **nephrologist** would consider as the primary indicator for renal replacement therapy in this patient?
Similarly, if you are a **nephrologist**, which variable do you think an **intensivist** would consider as the primary indicator for renal replacement therapy in this patient?
Please select only one of the following options:
○ He/she would start renal replacement therapy immediately
○ Serum potassium
○ Oliguria
○ pH
○ Positive fluid balance
○ Serum urea
○ Uremia symptoms

**Designating collaborators**

[ ] Designate a nephrologist from your institution to respond this survey.
**Answer this question only if:**
You responded “ICU chair” *or* “ICU physician (regular work schedule)” to question '1 [type]' (What is your function in the unit?) Please write your answer(s) here:

Name:

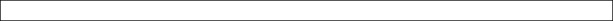

E-mail:

Mobile:

[ ] Designate one of the regular attending physicians at the unit to respond the questionnaire; if there is no regular attending physician at the unit, please name a colleague with long working hours at the unit.
**Answer this question only if:**
You responded “ICU chair” *or* “ICU physician (regular work schedule)” to question '1 [type]' (What is your function in the unit?) Please write your answer(s) here:

Name:

E-mail:

Mobile:

Submit the questionnaire.

Thank you for completing the questionnaire.
